# Recurrence of suicidal episodes in individuals with probable borderline personality disorder: one-year follow-up study

**DOI:** 10.3389/fpsyt.2026.1913088

**Published:** 2026-09-30

**Authors:** Lucía Albarracín-García, Laura Jiménez-Muñoz, Carmen Artés, Carmen Aguilar-Castillo, Laura Navío-García, Paula García-Barja, Olatz Lopez-Fernandez, Enrique Baca-García

**Affiliations:** 1 Department of Psychiatry, Hospital Universitario Fundación Jiménez Díaz, Madrid, Spain; 2 Department of Psychiatry, Hospital Universitario Rey Juan Carlos, Móstoles, Spain; 3 Department of Psychiatry, Hospital Universitario General de Villalba, Madrid, Spain; 4 Department of Psychiatry, Hospital Universitario Infanta Elena, Valdemoro, Spain; 5 Universidad Alfonso X el Sabio (UAX), Facultad de Psicología, Madrid, Spain; 6 Department of Psychiatry, Universidad Autónoma de Madrid, Madrid, Spain; 7 Instituto de Investigación Sanitaria de la Fundación Jiménez Díaz, Madrid, Spain; 8 Universidad Nacional de Educación a Distancia, Madrid, Spain; 9 Department of Psychology, Universidad Catolica del Maule, Talca, Chile; 10 CIBERSAM, ISCIII (Biomedical Research Networking Centre for Mental Health), Madrid, Spain

**Keywords:** anxiety, borderline personality disorder, depression, psychiatric comorbidity, suicide attempt

## Abstract

**Background:**

Borderline personality disorder (BPD) is strongly associated with suicidal behavior and high rates of reattempts. Short-term prediction of recurrent emergency presentations after a suicidal crisis in individuals with BPD traits remains clinically challenging, despite extensive study of suicidality in BPD more broadly. This study examined sociodemographic, clinical, and psychiatric predictors of suicidal recurrence and time to recurrence over 12 months in individuals with probable BPD presenting with suicidal behavior.

**Methods:**

A prospective cohort study enrolled adults with probable BPD — defined as meeting the validated MSIBPD screening cut-off (≥7) and/or having a pre-existing formal clinical diagnosis of BPD documented in medical records — with recent suicidal behavior from psychiatric emergency departments across four hospitals in Madrid, Spain. Participants were followed for 12 months and classified as recurrent or non-recurrent based on new suicidal behavior requiring emergency or hospital care within the participating hospital network. Group comparisons used chi-square tests and analysis of variance. Recurrence was estimated with Kaplan–Meier analyses. A multivariable Cox model assessed predictors including baseline depressive severity, lifetime mood disorders, lifetime anxiety-related disorders, and their interaction.

**Results:**

A total of 266 participants were included (mean age 31.2 years; 70% female). Seventy-six participants (28.6%) experienced recurrent suicidal behavior; the Kaplan–Meier-estimated 12-month recurrence rate was 28.6% (95%CI 23.1–34.0%), closely matching the crude proportion since no participant was censored before completing the full 12-month observation window. Sociodemographic characteristics were not associated with recurrence. Higher baseline depressive severity was independently associated with increased recurrence hazard (hazard ratio [HR] 1.03, 95%CI 1.01–1.05; p=0.003). Lifetime anxiety-related disorders were not independently associated with recurrence, and lifetime mood disorders showed a non-significant trend (HR 0.23; p=0.061). A statistically significant, though imprecisely estimated, interaction between lifetime mood and anxiety-related disorders was observed (HR 6.66, 95%CI 1.35–32.71; p=0.020).

**Conclusions:**

Recurrent suicidal behavior among individuals with probable BPD was associated with acute depressive severity and with an imprecisely estimated interaction between lifetime mood and anxiety-related disorders, independent of sociodemographic factors. These hypothesis-generating findings support closer monitoring of depressive symptoms and affective-anxiety comorbidity as part of post-crisis risk stratification, pending replication in larger, prospectively diagnosed samples.

**Trial registration:**

NCT04775160.

## 1 Introduction

Suicide constitutes one of the leading causes of years of life lost worldwide and remains a major public health priority. In 2021, approximately 720,000 deaths by suicide were estimated globally, ranking as the third leading cause of death among individuals aged 15–29 years (1, 2). Beyond its human cost, suicide imposes a substantial economic and social burden. A recent study conducted in France estimated the total cost associated with suicides and suicide attempts at €18.5 billion in 2019, including direct healthcare expenditures, productivity losses, and other indirect costs (3). Together, these figures underscore the urgent need to strengthen both primary and secondary suicide prevention strategies.

In Spain, suicidal behavior represents a major public health concern, with increasing rates of emergency department visits due to suicide attempts in recent years. Large-scale studies have reported substantial clinical burden and high recurrence rates among psychiatric emergency users, highlighting the need for improved secondary prevention strategies in real-world clinical settings (4). In line with this, previous Spanish cohort studies have examined predictors of suicide reattempts using survival approaches, underscoring the relevance of longitudinal designs in this context (5).

A central clinical challenge in suicide prevention is the recurrence of suicide attempts, as a prior attempt remains the strongest predictor of future suicidal behavior (6). Numerous studies have reported high rates of repetition across diverse clinical populations, highlighting the persistent vulnerability that follows an initial attempt (7). Evidence consistently indicates that a substantial proportion of individuals who attempt suicide will reattempt within relatively short follow-up periods, emphasizing the importance of timely and sustained preventive interventions (8, 9). Notably, the risk of recurrence is particularly elevated during the months immediately following an initial attempt, with approximately 70% of reattempts occurring during this critical period (10).

Psychiatric disorders constitute one of the most robustly recognized risk factors for suicidal behavior. Autopsy studies have reported that up to 90–95% of individuals who die by suicide present with underlying mental health problems (11). Within the spectrum of suicidal behavior, individuals who make multiple suicide attempts exhibit a more severe clinical profile, characterized by higher rates of anxiety disorders, severe depression, alcohol and substance misuse, aggressiveness, and hopelessness (12). In this context, Cluster B personality disorders—including borderline personality disorder (BPD)—have been independently associated with an increased risk of recurrent suicide attempts during follow-up (5).

Borderline personality disorder is characterized by emotional instability, impulsivity, identity disturbance, and unstable interpersonal relationships, and is frequently accompanied by self-harming behaviors (13). The prevalence of BPD in the general population ranges from 1% to 6%, while in clinical settings it may exceed 20% (14). Individuals with BPD present a markedly elevated risk of suicidal behavior. Reviews and prospective studies indicate that between 40% and 85% have made at least one suicide attempt, and between 3% and 10% die by suicide—rates that are substantially higher than those observed in the general population (15–17).

Suicidal risk in BPD is further shaped by a combination of clinical, psychosocial, and neurobiological factors. These include impulsivity, intense emotional distress, comorbidity with major depressive disorder, bipolar disorder, and substance use disorders, as well as alterations in frontolimbic brain regions implicated in emotional regulation (18). Additional risk factors include a history of childhood sexual abuse (19), poor psychosocial functioning, and broader indicators of psychosocial impairment (20). Longitudinal evidence suggests that, even after controlling for relevant clinical and demographic variables—such as female sex, low socioeconomic status, substance misuse, and traumatic experiences including childhood sexual abuse and post-traumatic stress disorder—specific core features of BPD, including identity disturbance, chronic feelings of emptiness, and frantic efforts to avoid abandonment, remain significantly associated with subsequent suicide attempts (21). Nevertheless, findings across studies remain heterogeneous, and the independent predictive value of BPD for suicide reattempts is not fully consistent across samples and methodological designs. Early empirical work by Soloff et al. (17) contributed to identifying key clinical risk factors for suicidal behavior in BPD.

In this context, BPD is consistently associated with suicidal behaviors (SB) and high rates of recurrence. Short-term prediction of who will re-present after an index crisis, however, remains clinically challenging: the risk of suicidal recurrence in this population does not appear to be solely attributable to the presence of BPD traits, but is likely influenced by the interplay of multiple clinical and psychosocial factors, particularly lifetime psychiatric comorbidity and the severity of depressive symptoms. Despite the high prevalence of these factors among individuals with BPD traits, their combined prognostic value for suicidal recurrence remains insufficiently characterized in clinical samples.

Therefore, the aim of the present study is to examine sociodemographic, clinical, and psychiatric comorbidity predictors, as well as variables related to time to suicidal recurrence, over a 12-month follow-up period in a clinical sample of individuals with borderline personality traits and SB. This study is intended as an exploratory, hypothesis-generating contribution to short-term post-crisis risk stratification in this population, rather than as the identification of a novel BPD-specific suicidal phenotype.

## 2 Methods

### 2.1 Study design

A prospective cohort study was conducted with a 12-month follow-up after an index suicidal episode (suicide attempt, suicidal ideation, or non-suicidal self-injury; see Section 2.2 for the exact eligibility criteria and Section 3.1 for the observed composition of the sample by index episode type). This study is an observational secondary analysis conducted within the SmartCrisis research infrastructure, which evaluates mobile monitoring interventions and digital safety plans for secondary suicide prevention (22, 23). It does not evaluate the effect of the SmartCrisis mobile-monitoring intervention or digital safety plan on recurrence; intervention-arm assignment was not modeled as a predictor in the present analysis, and readers are referred to (23, 24) for trial-specific intervention-effect analyses. The full protocol of the SmartCrisis 2.0 randomized controlled trial was published by Barrigon and colleagues (24). Data collection took place from 2022 to 2024, and participants were followed until the end of 2025.

### 2.2 Participant sample

The sample consisted of adults with probable BPD (meeting the MSI-BPD ≥7 screening cut-off and/or a pre-existing formal clinical diagnosis of BPD in medical records) and suicidal behavior recruited from psychiatric emergency units and mental health services at four hospitals in Madrid (Spain): Hospital Universitario Fundación Jiménez Díaz, Hospital Universitario General de Villalba, Hospital Universitario Rey Juan Carlos, and Hospital Universitario Infanta Elena. Consecutive eligible patients presenting to the participating emergency departments during recruitment coverage hours were approached for enrollment. After follow-up, participants were classified according to the occurrence of new suicidal episodes during the 12-month period as non-recurrent or recurrent.

Inclusion criteria are:

- A qualifying index suicidal episode within the month prior to recruitment, defined as a suicide attempt, an episode of suicidal ideation, or an act of non-suicidal self-injury (NSSI) prompting emergency or psychiatric evaluation. In the enrolled sample, this qualifying episode was a suicide attempt for 149 participants (56.0%), suicidal ideation for 92 (34.6%), and NSSI for 25 (9.4%); the type of qualifying episode was not associated with subsequent 12-month recurrence status (χ²(df=2)=0.42, p=.809; see **Table 1**).
- Age ≥ 18 years.
- Capacity to provide informed consent.
- Availability of a smartphone for the mobile follow-up application.

**Table 1:** Baseline sociodemographic and clinical characteristics by recurrence status (N = 266).

Variable	Total (N = 266)	Non-recurrent (n=190)	Recurrent (n=76)	Test statistic	p-value
Age, mean (SD), years	31.5	31.8	31.0	F=0.43	.513
Female sex, n (%)	191 (71.8%)	137 (72.1%)	54 (71.1%)	χ²=0.00	1.00
Immigration status, n (%)	81 (30.5%)	56 (29.5%)	25 (32.9%)	χ²=0.16	.689
Educational level, n (%)				χ²=1.08	.898
*None/primary*	2 (0.8%)	2 (1.1%)	0 (0%)		
*Secondary*	186 (69.9%)	134 (70.5%)	52 (68.4%)		
*Bachillerato (upper secondary)*	15 (5.6%)	10 (5.3%)	5 (6.6%)		
*University*	63 (23.7%)	44 (23.2%)	19 (25.0%)		
Current employment status, n (%)				χ²=9.09	.105
*Active/student*	108 (40.6%)	81 (42.6%)	27 (35.5%)		
*Unemployed (with or without benefit)*	66 (24.8%)	49 (25.8%)	17 (22.4%)		
*Disability (permanent or temporary)*	88 (33.1%)	58 (30.5%)	30 (39.5%)		
*Retired*	4 (1.5%)	2 (1.1%)	2 (2.6%)		
Household income level, n (%)				χ²=1.13	.890
*<€500/month*	17 (6.4%)	12 (6.3%)	5 (6.6%)		
*€500–1,499/month*	89 (33.5%)	63 (33.2%)	26 (34.2%)		
*€1,500–1,999/month*	45 (16.9%)	30 (15.8%)	15 (19.7%)		
*€2,000–2,499/month*	41 (15.4%)	31 (16.3%)	10 (13.2%)		
*>€2,500/month*	66 (24.8%)	49 (25.8%)	17 (22.4%)		
Marital/family status, n (%)				χ²=6.44	.169
*Single*	180 (67.7%)	132 (69.5%)	48 (63.2%)		
*Married/cohabiting>6 months*	43 (16.2%)	28 (14.7%)	15 (19.7%)		
*Separated/divorced/widowed*	40 (15.0%)	30 (15.8%)	10 (13.2%)		
*Unknown/not reported*	2 (0.8%)	0 (0%)	2 (2.6%)		
Qualifying index episode: attempt/ideation/NSSI, n (%)				χ²=0.42	.809
*Suicide attempt*	149 (56.0%)	107 (56.3%)	42 (55.3%)		
*Suicidal ideation*	92 (34.6%)	64 (33.7%)	28 (36.8%)		
*Non-suicidal self-injury (NSSI)*	25 (9.4%)	19 (10.0%)	6 (7.9%)		
Lifetime anxiety-related disorders (F4), n (%)	188 (70.7%)	68.3%	82.9%	χ²=5.74	.022
Lifetime schizophrenia-spectrum/psychotic disorders (F2), n (%)	4 (1.5%)	0.5%	3.9%	χ²=4.08	.042
Lifetime mood disorders (F3), n (%)	107 (40.2%)	39.3%	46.1%	χ²=0.74	.390
Lifetime major depressive disorder, n (%)	21 (7.9%)	9.8%	3.9%	χ²=1.77	.183
Substance use, eating disorders, PTSD (lifetime)	16 (6.0%)	6.0%	6.6%	χ²=0.00	1.00

Sex, immigration status, educational level, current employment status, household income, marital/family status, and lifetime F2/F3/F4 psychiatric comorbidity were computed directly from the analytic dataset (N = 266: 190 non-recurrent, 76 recurrent).

Whereas patients will be excluded due to severe neurological or cognitive disorders, and inability to complete clinical or digital follow-up.

### 2.3 Instruments

The Columbia–Suicide Severity Rating Scale (C-SSRS) is a semi-structured instrument designed to assess suicidal ideation and behavior, including intensity, frequency, and lethality across time periods (25). The scale has demonstrated strong predictive validity for future suicide attempts and has been widely adopted in both clinical and research settings. A Spanish-adapted version has been used in clinical populations, showing adequate reliability and validity for the assessment of suicidal behavior in Spanish-speaking contexts.

The MSI-BPD is a 10-item self-report screening tool designed to identify borderline personality disorder traits. A score ≥7 indicates a high probability of BPD, while scores of 5–6 suggest the need for further diagnostic evaluation. The Spanish version of the MSI-BPD has been validated in clinical and non-clinical Spanish-speaking samples, demonstrating good psychometric properties, including high internal consistency (KR-20 = 0.873) and adequate test–retest reliability (ICC = 0.87). The scale also shows satisfactory discriminant validity, with good sensitivity (0.71) and specificity (0.68) for detecting BPD when using the established cut-off score (26). In this study, BPD status was operationalized as meeting either the validated MSI-BPD screening cut-off (≥7) or a pre-existing formal clinical diagnosis of BPD documented in medical records. Of the 266 participants in the analytic sample, 236 (88.7%) met the MSI-BPD cut-off and 92 (34.6%) had a documented clinical diagnosis of BPD in medical records, with every participant meeting at least one of the two criteria (62 participants met both). This dual ascertainment strengthens case definition relative to screening alone; however, the majority of participants (174/266, 65.4%) were included on the basis of the MSI-BPD screening cut-off without independent structured-interview confirmation of a categorical diagnosis. Participants are therefore best characterized throughout this manuscript as having probable BPD (screening-positive and/or clinically diagnosed) rather than as having a diagnosis uniformly confirmed through a structured diagnostic interview.

Other clinical and sociodemographic variables: Depression severity was assessed using the Inventory of Depressive Symptomatology (IDS), a clinician- and self-report measure of depressive symptoms with excellent internal consistency (α > 0.90) and strong convergent validity with other measures of depression severity (27, 28). Insomnia severity was measured using the Insomnia Severity Index (ISI), a brief self-report instrument with good internal consistency (α ≈ 0.80–0.90) and strong construct validity (29), with a validated Spanish version showing adequate psychometric properties in clinical samples (30). Health-related quality of life was assessed using the EQ-5D-5L, a standardized measure of health status widely used in clinical research (31). The Spanish value set was applied following EuroQol methodology (32, 33). No dimensional instrument for current anxiety symptoms (e.g., a current-anxiety scale such as the STAI or HAM-A) was administered in this study.

Psychiatric comorbidity was classified according to ICD-10 diagnostic criteria using structured clinical records, following standardized procedures established in the SmartCrisis protocol (22, 24). The ICD-10 F3 (lifetime mood disorders) category used in the multivariable model is a composite drawn from structured clinical records and encompasses unipolar depressive and bipolar-spectrum presentations; a dedicated dimensional screening instrument for hypomanic, cyclothymic, or mixed-affective symptoms was not administered. Anxiety-related comorbidity (ICD-10 F4) was likewise captured exclusively through lifetime categorical diagnoses drawn from structured clinical records, not through a dimensional measure of current anxiety severity, anxious arousal, or anxiety sensitivity.

### 2.4 Procedure

At the baseline assessment, a licensed clinical psychologist collected sociodemographic data and administered the C-SSRS together with the MSI-BPD screening scale. Baseline assessment was conducted after clinical stabilization of the index suicidal crisis — typically prior to discharge from the emergency department or during short-stay observation, and in a minority of cases at a subsequent outpatient visit — and always after the participant was judged to have the capacity to provide informed consent, consistent with the inclusion criteria; it was not conducted during the acute emergency presentation itself.

The primary outcome, recurrent suicidal behavior, was defined as any new presentation to emergency department or hospital services within the 12-month follow-up period, coded as a suicide attempt according to ICD-10 diagnostic codes and classified using the standardized ICD-10 suicidality phenotype definition of Monson et al. (34). This single term is used consistently throughout the manuscript; “index suicidal behavior” refers exclusively to the qualifying baseline event.

To ascertain the occurrence of suicidal behavior during follow-up, emergency department and hospitalization records were systematically reviewed. All emergency care records were coded by trained clinical coders using the International Classification of Diseases, Tenth Revision (ICD-10). Suicidal behavior was identified based on ICD-10 diagnostic codes and subsequently classified according to a standardized suicidality phenotype definition (34). Recurrence was ascertained exclusively through this systematic review of records at the four participating hospitals; participants were not independently re-contacted for a structured 12-month outcome interview outside of this record review. Suicidal or self-harming behavior managed outside this hospital network, in outpatient settings not requiring emergency care, or at home without medical contact, would therefore not have been captured, and the reported recurrence rate should be interpreted as the rate of recurrence requiring emergency/hospital care within the participating network rather than the rate of all suicidal behavior. This approach allowed for the objective verification of suicide reattempts during the follow-up period and ensured consistency in outcome assessment across clinical settings.

#### 2.4.1 Ethical considerations and data privacy

Eligible patients received a detailed explanation of the study and provided written informed consent prior to enrolment. Participation was voluntary and no monetary incentives were offered. Data were anonymized by assigning each participant a coded identifier, and confidentiality was ensured through restricted access to any re-identification information. Losses to follow-up and missing data were systematically recorded following standardized procedures, and all evaluators received specific training to ensure consistency in data collection and reliability of assessments.

The study complied with the principles of the Declaration of Helsinki and was approved by the Ethics Committee of the University Hospital Fundación Jiménez Díaz, Reference number EC005-21-FJD. Data handling complied with the General Data Protection Regulation (EU 2016/679) and applicable Spanish legislation on personal data protection and patient rights.

### 2.5 Statistical analysis

Descriptive analyses were conducted for sociodemographic and clinical variables. Group comparisons between recurrent and non-recurrent participants were performed using chi-square tests for categorical variables and analysis of variance (ANOVA) for continuous variables, as appropriate.

The primary outcome was time to first suicide reattempt during the 12-month follow-up period. All 190 non-recurrent participants completed the full 12-month (365-day) observation window without being lost to follow-up at an earlier time point, and no deaths were recorded during follow-up; censoring in the Kaplan–Meier and Cox analyses therefore occurred exclusively at the administrative end of the 12-month follow-up window, and “last available contact” for censored participants corresponds operationally to this 12-month endpoint rather than to a variable, participant-specific loss-to-follow-up date. A multivariable Cox proportional hazards survival model was applied, including current occupational status, insomnia severity (ISI), depression severity (IDS), quality of life (EQ-5D-5L), and lifetime psychiatric comorbidity according to ICD-10 as covariates. Variables were initially selected based on clinical relevance; a backward stepwise Cox regression approach, retaining terms significant at p<.05, was then applied to derive the final model, with the F3×F4 interaction term retained *a priori* given its clinical relevance and specified in advance of the stepwise selection. The proportional-hazards assumption for the final model was assessed using Schoenfeld residual correlations with survival time for each covariate; no covariate showed a statistically significant time-dependent effect (all p>.14), supporting the proportional-hazards assumption for the reported model. Sample size was not determined *a priori* via a dedicated power calculation for this analysis; it reflects the number of participants enrolled in the SmartCrisis 2.0 program (2022–2024) who met eligibility criteria for the present sub-study. The final multivariable Cox model included 257 participants and 76 recurrence events (approximately 19 events per covariate term), which we consider adequate to limit overfitting risk given the number of model terms. Nine of 266 participants (3.4%) were excluded from the multivariable Cox model due to missing data on one or more covariates; a complete-case analysis approach was used for the primary model. A sensitivity analysis excluding the five participants with a lifetime bipolar-spectrum diagnosis yielded materially unchanged estimates (F3×F4 interaction HR = 6.31, 95% CI 1.28–30.98, p=.023, versus HR = 6.66, 95% CI 1.35–32.71, p=.020 in the full sample), indicating that the interaction finding is not driven by this small subgroup. As a secondary, exploratory analysis complementing the primary Cox model, a multivariable logistic regression model was fitted with 12-month recurrence status (yes/no) as the outcome, including the same four terms as the primary Cox model (IDS-C, lifetime F3, lifetime F4, and their interaction). All analyses were conducted using JAMOVI 2.7), accounting for missing data management and adjustment for potential confounders, in accordance with the SmartCrisis protocols.

## 3 Results

### 3.1 Baseline characteristics

Of the 266 participants with probable BPD included in the 12-month follow-up analysis, 190 (71.4%) did not experience recurrent suicidal behavior, while 76 (28.6%) presented to emergency services for subsequent suicidal behavior episodes.

A full baseline characteristics table (**Table 1**), reporting the total sample, recurrent and non-recurrent groups, test statistics, p-values, missingness, and effect sizes for all sociodemographic, clinical, and psychiatric comorbidity variables, is presented below and precedes the Cox regression table (**Table 2**).

**Table 2:** Multivariable Cox proportional hazards model for suicidal recurrence (N = 257) — previously “**Table 1**” in the original submission; content unchanged.

Variable	Hazard ratio (HR)	95% CI for HR	p-value
Baseline Depressive Severity (IDS-C)	1.032	[1.011, 1.054]	.003
Lifetime Mood Disorder (F3)	0.234	[0.051, 1.067]	.061
Lifetime Anxiety Disorder (F4)	1.072	[0.534, 2.150]	.846
F3 x F4 Interaction	6.655	[1.354, 32.705]	.020

HR, hazard ratio; CI, confidence interval. Model fit: AIC = 803.33; BIC = 812.66. Total events recorded: 76. Nine observations were excluded due to missing data.

No significant differences were observed between groups regarding sociodemographic variables, including median age (31.0 vs. 31.8 years; F(df=1,264)=0.43,p=.513), gender distribution (χ²(df=1)=0.00,p=1.00), or immigration status (p=.582). Similarly, educational level, income distribution, and current employment activity showed no significant associations with suicidal recurrence (all p>.10). Regarding social support and living arrangements, groups were comparable in terms of marital status (p=.172) and household composition, including living with parents, children, or partners (all p>.40).

The type of qualifying index episode (suicide attempt, suicidal ideation, or non-suicidal self-injury) was similarly distributed between the non-recurrent and recurrent groups (χ²(df=2)=0.42,p=.809). Suicide attempts were the most frequent qualifying episode in both groups (56.3% and 55.3%, respectively), followed by suicidal ideation (33.7% and 36.8%) and NSSI (10.0% and 7.9%).

To confirm that the type of qualifying index episode did not confound the primary survival analysis, we conducted a sensitivity analysis adding index episode type (suicidal ideation and NSSI, versus suicide attempt as reference) to the primary Cox model. Point estimates for baseline depressive severity, lifetime F3, lifetime F4, and the F3×F4 interaction were materially unchanged, and a likelihood ratio test indicated no improvement in model fit from adding index episode type (χ²(df=2)=0.23,p=.891); index episode type itself was not independently associated with recurrence (ideation vs. attempt: HR = 0.90,95%CI:0.55–1.47,p=.670; NSSI vs. attempt: HR = 0.88,95%CI:0.37–2.06,p=.760). The same pattern was observed in the corresponding logistic regression model (likelihood ratio test χ²(df=2)=0.61,p=.739). The type of qualifying index episode was therefore not retained in the final multivariable model.

Analysis of lifetime psychiatric disorders (ICD-10 categories) revealed significant differences in two areas. Participants in the recurrent group exhibited a significantly higher prevalence of lifetime anxiety, obsessive-compulsive, and stress-related disorders (F4) compared to the non-recurrent group (82.9% vs. 68.3%; χ2(df=1)=5.74,p=.022;OR=2.35,95%CI:1.20–4.89) in this unadjusted comparison; however, F4 alone did not remain independently significant once the F3×F4 interaction term was included in a multivariable model (see Section 3.2 and **Table 2**). Additionally, lifetime schizophrenia spectrum and psychotic disorders (F2) were more frequent among recurrent cases (3.9% vs. 0.5%; χ2(df=1)=4.08,p=.042), although this association did not reach significance in multivariable models.

Lifetime mood disorders (F3) and major depressive disorder did not show independent significant associations with recurrence (p=.322 and p=.112, respectively). No other psychiatric comorbidities, including substance use, eating disorders, or PTSD, reached statistical significance (all p>.10).

Among participants with available lifetime diagnostic records, unipolar major depressive disorder was recorded for 21 (8.1%) and a bipolar-spectrum diagnosis for 5 (1.9%); bipolar-spectrum presentations were therefore infrequent in this sample. The composition of the four F3×F4 comorbidity subgroups and their recurrence rates were: no F3/no F4, n=45 (11 recurrence events, 24.4%); F4 only (no F3), n=107 (30 events, 28.0%); F3 only (no F4), n=26 (2 events, 7.7%); both F3 and F4, n=81 (33 events, 40.7%) (**Table 3**). These four subgroups sum to 259 participants; the remaining 7 participants had missing lifetime F3/F4 diagnostic status and are excluded from this subgroup breakdown (all 7 were non-recurrent). The recurrence rate across the 259 participants with complete F3/F4 data (76/259, 29.3%) is closely comparable to the recurrence rate reported for the full cohort (76/266, 28.6%). The comparatively small number of events observed in the “F3 only” subgroup (n=26, 2 events) contributes to the wide confidence interval around the F3×F4 interaction term reported below.

**Table 3:** Composition and recurrence rate of the four F3×F4 lifetime comorbidity subgroups, analytic dataset (N = 259 with complete lifetime F3/F4 status, of N = 266 in the full cohort).

Subgroup	N	%	Recurrence events	Recurrence rate
No F3/No F4	45	17.4%	11	24.4%
F4 only (no F3)	107	41.3%	30	28.0%
F3 only (no F4)	26	10.0%	2	7.7%
F3 and F4	81	31.3%	33	40.7%
Total	259	100%	76	29.3%

New table, added in response to reviewer comments.

### 3.2 Survival analysis

In univariate Kaplan–Meier analyses, recurrence remained high during the first year following the index emergency department visit (**Figure 1**). The 12-month recurrence rate for the cohort was 28.6% (95% CI: 23.1%-34.0%).

**Figure 1:**
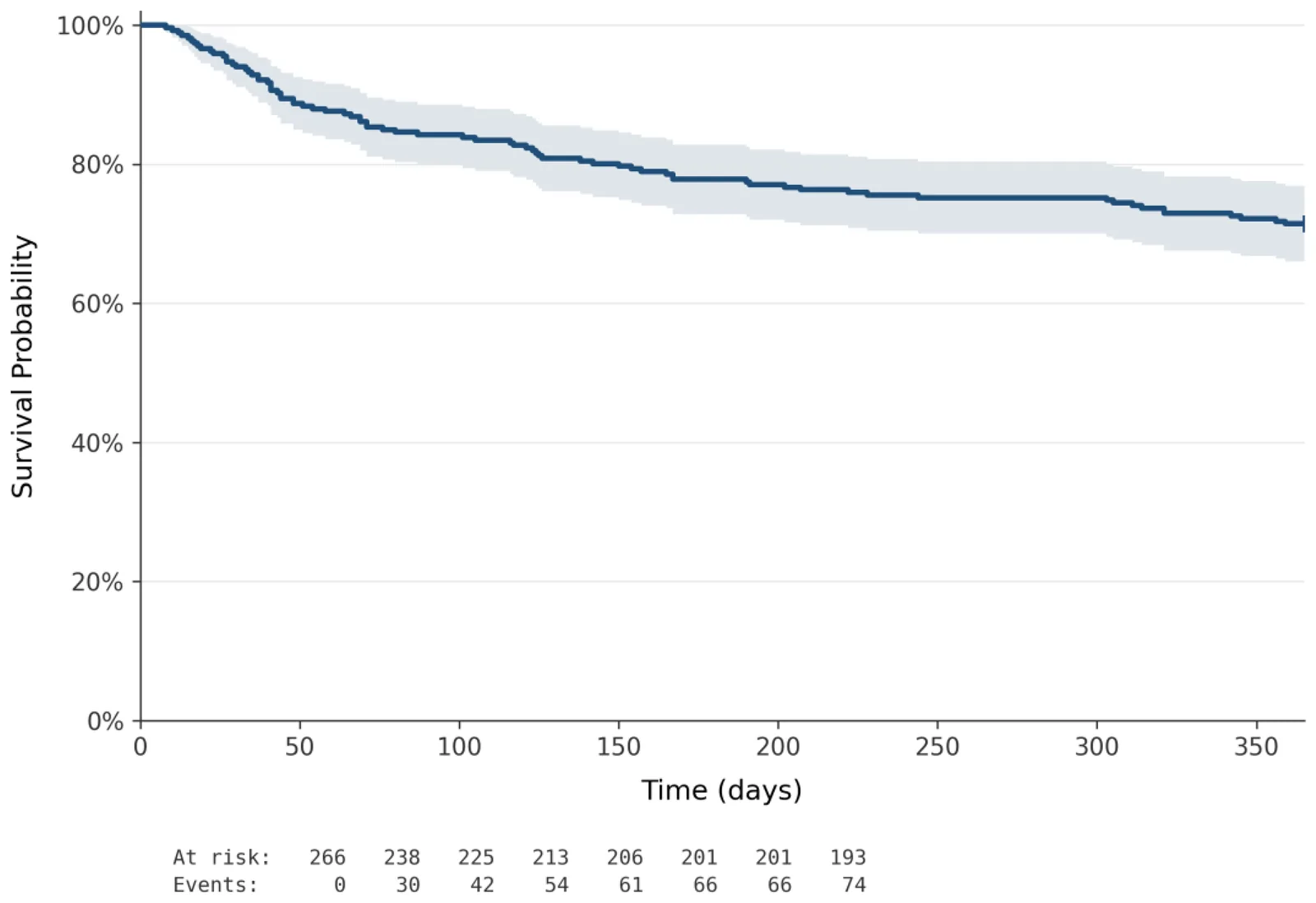
Kaplan–Meier curve for suicide reattempt within 12 months of index episode.

To identify independent predictors of time to recurrent suicidal behavior, a multivariable Cox proportional hazards model was constructed. The model included baseline depressive severity (IDS–C total scores), lifetime mood disorders (F3), lifetime anxiety-related disorders (F4), and the interaction term between lifetime F3 and F4.

Baseline depressive severity (IDS-C) was significantly associated with the hazard of recurrence (HR = 1.032,95%CI:1.011–1.054,p=.003). Regarding lifetime diagnoses, anxiety-related disorders (F4) showed no independent association with time to recurrence (HR = 1.072,95%CI:0.534–2.150,p=.846), while mood disorders (F3) showed a non-significant trend (HR = 0.234,95%CI:0.051–1.067,p=.061). A statistically significant, though imprecisely estimated, interaction was observed between lifetime F4 and F3 disorders (HR = 6.655,95%CI:1.354–32.705,p=.020); this estimate is drawn from a comparatively small number of events in the “F3 only” subgroup (n=26, 2 events) relative to the other three F3×F4 subgroups, and should be considered exploratory pending replication in a larger sample with more events. The estimates and hazard ratios for the full model are presented in **Table 2**, **Figure 2**.

**Figure 2:**
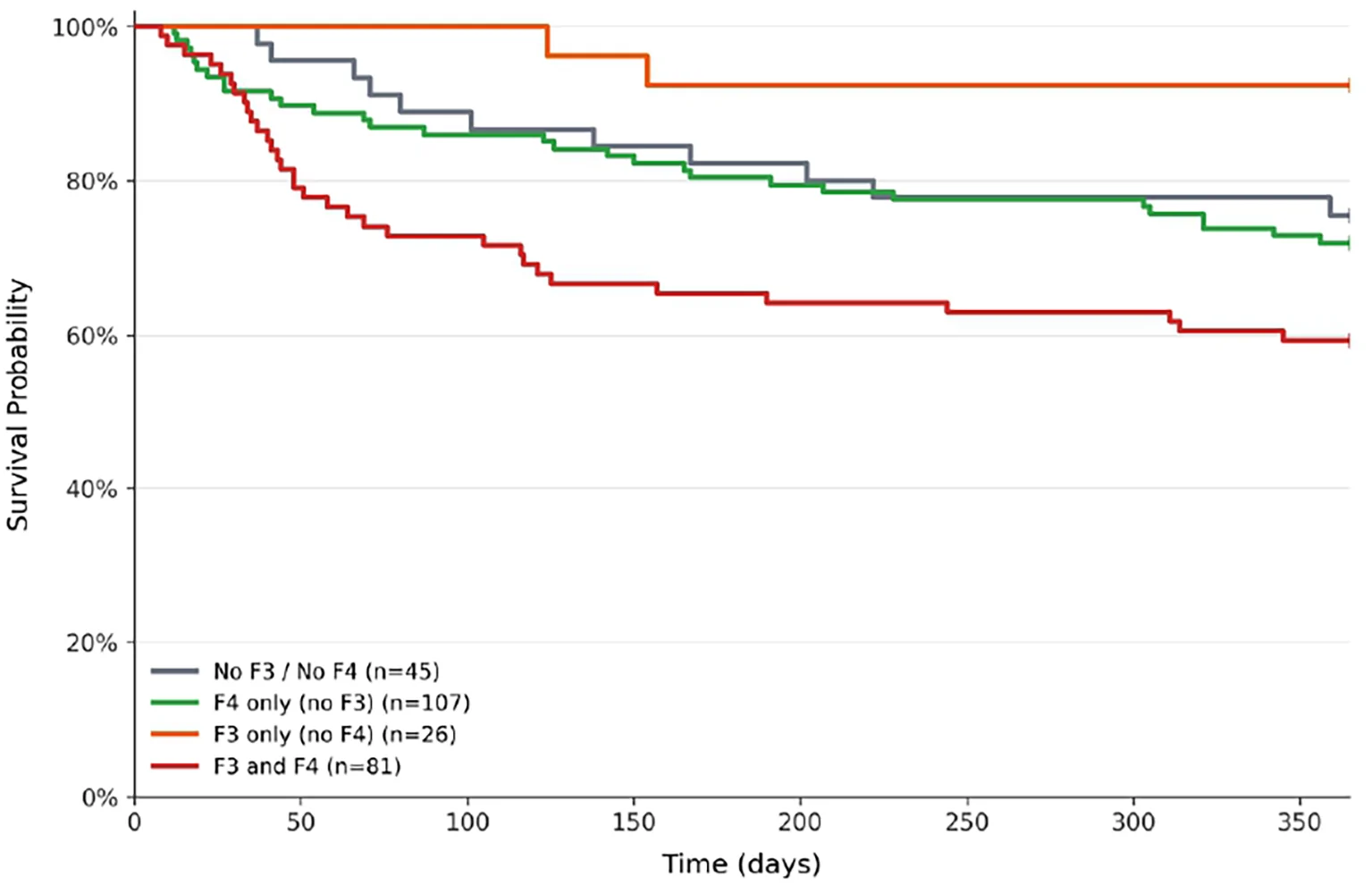
Kaplan–Meier curves for recurrent suicidal behavior, stratified by the four F3×F4 comorbidity subgroups (no F3/no F4, n=45; F4 only, n=107; F3 only, n=26; F3 and F4, n=81).

As a secondary, exploratory analysis, a multivariable logistic regression model was fitted with 12-month recurrence status (yes/no) as the outcome, including the same four terms as the primary Cox model. Consistent with the survival analysis, baseline depressive severity (IDS-C; aOR=1.04, 95% CI 1.01–1.07, p=.005) and the F3×F4 interaction (aOR=7.99, 95% CI 1.40–45.43, p=.019) were independently associated with 12-month recurrence, while lifetime F4 alone (aOR=1.07, 95% CI 0.47–2.41, p=.880) and lifetime F3 alone (aOR=0.20, 95% CI 0.04–1.03, p=.055) were not independently significant once the interaction term was included. This pattern mirrors the primary Cox model and reinforces that it is the co-occurrence of lifetime mood and anxiety-related disorders, rather than either alone, that is associated with recurrence risk; this finding is presented in addition to, and should be interpreted alongside, the primary time-to-event Cox model reported above, given the different outcome definitions (binary recurrence status vs. time to recurrence) and analytic assumptions of the two approaches.

## 4 Discussion

In this prospective cohort study of individuals with probable borderline personality disorder presenting with suicidal behavior, we aimed to identify predictors of recurrence over a 12-month follow-up period. Despite extensive research, the population of individuals with BPD traits most at risk of suicidality remains hard to characterize and identify for preventive care (35). This study provides exploratory evidence supporting the relevance of acute depressive severity and lifetime affective-anxiety comorbidity in post-crisis risk stratification, rather than establishing a novel BPD-specific suicidal phenotype. Sociodemographic factors did not significantly differentiate those who recurred and those who did not. A statistically significant, though imprecisely estimated, interaction between lifetime anxiety and mood disorders, alongside baseline depressive severity, was the strongest predictor of time to recurrence (HR 6.66, 95% CI 1.35–32.71; p = 0.020). These results suggest that the combined burden of lifetime comorbid anxiety and mood disorders and acute depressive severity is associated with suicidal recurrence in this population, though replication in larger samples with more events is needed before this can be regarded as a specific or robust clinical phenotype.

Co-occurrence of suicidal behavior and BPD has been widely documented (21, 36). Our study found a 12-month recurrence rate of 28.6%, underscoring the high acuity of individuals with probable BPD following an emergency presentation. This pattern aligns with findings from clinical studies conducted in Spain, which show that individuals with personality disorders frequently present with medically serious suicide attempts and complex clinical profiles in psychiatric emergency settings (37).

Psychological autopsy studies similarly indicate that personality disorders, particularly borderline personality disorder, are highly prevalent among individuals who die by suicide, often co-occurring with affective and anxiety disorders. Collectively, this body of evidence supports the view that suicidal behavior in BPD is best conceptualized as the result of interacting psychopathological dimensions rather than a single diagnostic entity (38).

In line with this, studies in Spanish clinical samples have also identified significant differences in clinical severity and psychopathological characteristics between patients with BPD who present with suicide attempts or non-suicidal self-injury and those who do not (39, 40).

Differences in reported recurrence rates across studies likely reflect variation in outcome definitions (e.g., emergency/hospital-verified suicide attempts versus broader definitions of self-harm inclusive of non-suicidal self-injury), follow-up duration, and recruitment setting (psychiatric emergency departments in our study versus general treatment or community settings in others) (41). Our 28.6% rate is slightly higher than the 26% reported by Links et al. (42) and lower than some recurrence rates observed in longitudinal studies of borderline personality disorder; while some studies focus exclusively on suicidal behavior with clear intent to die, others include a broader spectrum of self-injurious behaviors such as non-suicidal self-injury (NSSI), reflecting a more comprehensive clinical picture of BPD-related self-harm. Yen et al. (21) reported a lower 10-year recurrence rate of 21%, which may be explained by differences in sampling, as their cohort was recruited from general treatment settings rather than individuals with prior suicidal behavior. These methodological differences limit direct comparability of point estimates across studies and should be considered when interpreting our 28.6% recurrence rate.

The most significant finding of our survival analysis was the prognostic value of baseline depressive severity (IDS-C scores) and the interaction between lifetime anxiety and mood disorders. While univariate analyses failed to show independent associations with recurrence, the multivariate Cox regression showed that recurrence risk was substantially higher when anxiety and mood disorders co-occur (HR 6.66, 95% CI 1.35–32.71; p = 0.020). As this is an observational study, baseline depressive severity should be regarded as a prognostic marker of recurrence rather than an established causal or directly modifiable risk factor; intervention studies targeting acute depressive symptoms in this population are needed to test the latter. It is important to note that anxiety-related comorbidity in this study was captured exclusively through lifetime categorical ICD-10 diagnoses drawn from clinical records, not through a dimensional measure of current anxiety symptoms, anxious arousal, or anxiety sensitivity; the interpretation offered here is therefore hypothesis-generating rather than directly supported by our measurement approach. Most survival studies in BPD have focused predominantly on depressive symptoms or stable personality traits, often overlooking the potential impact of anxiety disorders or failing to identify this comorbid association (42). While several studies have identified depression as a primary driver of suicidal behavior in BPD cohorts (43–45), our results offer a slight shift from recent findings: while Mirkovic and colleagues (46) identified historical mood disorder as a key predictor of recurrence, our model found historical mood disorders only gained predictive power through their interaction with anxiety, and current depressive severity at baseline (IDS-C) emerged as an independent predictor of risk. This distinction may be of clinical relevance: it suggests that, for individuals with probable BPD, acute dysphoria (as measured by the IDS-C) may act as a distinct, prognostic marker of risk, separate from the more stable trait of personality pathology or a distal psychiatric history. Consequently, clinical interventions should consider prioritizing the assessment and management of acute depressive symptoms—regardless of whether the patient meets full criteria for a major depressive episode—as part of a localized strategy for short-term risk stratification.

Our findings align with the work of Podlogar et al. (47), who identified depression and a lifetime history of anxiety-related diagnoses as key discriminants of high suicide risk in BPD-related samples. Similarly, Tucker et al. (48) found that while affective dysregulation and despondence directly predict ideation, certain maladaptive personality traits influence suicidality indirectly through anxiety sensitivity. Comtois and colleagues (49) found higher prevalences of anxiety and depression in patients with BPD compared to other personality disorders but did not measure suicidality. Whereas depressive symptoms have been identified as short-term predictors of suicidality, broader indicators of poor social and interpersonal adjustment have also been consistently associated with long-term risk in BPD populations (17). This underscores the importance of early intervention targeting affective and interpersonal dysregulation to reduce suicidality risk.

The absence of significant sociodemographic associations in this sample should be interpreted cautiously (41). Possible explanations include restricted variability within a clinically selected, high-risk emergency sample; limited statistical power to detect subgroup effects; the fact that all participants had already crossed a high-acuity emergency threshold, which may homogenize risk; and unmeasured confounding by trauma exposure, socioeconomic adversity, social instability, or differential treatment access. These findings should not be interpreted as indicating that psychosocial or sociodemographic factors are unimportant to suicidal recurrence in this population; for instance, Yen et al. (21) identified increased risk associated with lower socioeconomic status, limited education, female gender, and unemployment in BPD cohorts, and the higher prevalence of BPD in females—as documented by Bozzatello et al. (50) and Wedig et al. (43)—often influences these epidemiological trends. Age is another frequently cited predictor, with younger populations typically exhibiting higher rates of suicidality in BPD (6, 51); despite our cohort being predominantly composed of younger adults, we found no association between age and recurrence, consistent with evidence from longitudinal studies of suicide risk in BPD reporting inconsistent or attenuated effects of sociodemographic variables in acute clinical samples (42).

### 4.1 Clinical and research implications

From a clinical perspective, these results underscore the potential value of specialized, high-intensity follow-up protocols for suicide prevention in individuals with probable BPD exhibiting elevated depressive scores or lifetime anxious comorbidity. Our findings suggest that traditional monitoring may be insufficient for this high-risk phenotype; interventions focusing on emotion regulation, stress management (52, 53) and the intensive treatment of acute depressive symptoms—regardless of whether the patient meets the full threshold for a Major Depressive Episode—could be particularly beneficial in mitigating the hazard of recurrence.

Furthermore, the identification of the co-occurrence of lifetime anxiety and mood disorders provides a possible target for personalized clinical attention. Clinicians may consider prioritizing evidence-based treatments known to address both affective dysregulation and anxiety sensitivity, such as Dialectical Behavior Therapy (DBT) (16), which may provide the necessary stability to bridge the gap between emergency care and long-term recovery (54). These interventions were not tested in the present study; our findings support the use of baseline depressive severity and lifetime mood–anxiety comorbidity to identify individuals at higher short-term risk of recurrence, but do not themselves demonstrate that DBT or high-intensity follow-up prevent recurrence in this population. Prospective intervention trials are needed to test this directly.

### 4.2 Strengths and limitations

The strength of this study is its prospective cohort design of a large sample, which allowed for a direct evaluation of suicidal recurrence over a standardized 12-month period.

However, several limitations must be acknowledged. First, BPD status was operationalized as meeting either the MSI-BPD screening cut-off (≥7) or a pre-existing formal clinical diagnosis in medical records; while every participant met at least one of these two criteria, the majority (65.4%) were included on the screening criterion alone, without independent structured-interview confirmation of a categorical diagnosis, and participants are best characterized as having probable BPD rather than a uniformly confirmed categorical diagnosis. Second, recurrence was ascertained exclusively through systematic review of emergency department and hospitalization records at the four participating hospitals; participants were not independently re-contacted for a structured 12-month outcome interview, so suicidal or self-harming behavior managed outside this hospital network, in outpatient settings, or at home without medical contact would not have been captured, which may underestimate the true recurrence rate. We cannot exclude residual confounding by unmeasured clinical or psychosocial variables. Medication adherence was assessed for a subset of participants using the Morisky scale, but specific psychotropic medications prescribed and changes in pharmacological or psychotherapeutic treatment during follow-up were not systematically recorded, as treatment was delivered according to routine clinical practice without protocol-driven standardization. The reliability of lifetime psychiatric diagnoses drawn from structured clinical records, rather than research-grade structured interviews, may also vary across sites and raters. This study did not include validated measures of perceived social support, chronic psychosocial stress, or peer/interpersonal relationship problems, all of which have been implicated as risk factors for suicidal recurrence in individuals with BPD traits, limiting our ability to disentangle the specific contribution of affective-anxiety comorbidity from broader, unmeasured psychosocial adversity. Childhood trauma, sexual abuse, and cumulative psychosocial adversity are highly relevant to suicidal behavior in BPD.

Bipolar-spectrum disorders, cyclothymia, and mixed/hypomanic features were not systematically assessed with a dedicated instrument; although bipolar-spectrum diagnoses were infrequent in the available clinical records (1.9%), we cannot exclude a residual contribution of unassessed mixed-affective states to the F3×F4 interaction, which itself, while statistically significant, is estimated from a comparatively small number of events in one of the four F3×F4 subgroups (F3 only, no F4: n=26, 2 events), and should be considered exploratory pending replication in a larger sample with more events. Anxiety-related comorbidity was captured only through lifetime categorical diagnoses, not a dimensional measure of current anxiety symptoms.

The requirement for smartphone ownership as an eligibility criterion is unlikely to have excluded a large share of the general population, as an estimated >95% of Spanish households now have mobile phone access (INE, 2025), and the vast majority of mobile devices in use are smartphones. However, this requirement may still have introduced selection bias against the specific subgroup of individuals with lower digital access or socioeconomic resources — who are disproportionately represented among psychiatric emergency populations — potentially limiting the generalizability of our findings to the broader population of individuals with BPD traits and suicidal behavior. Finally, recurrence was defined by representation to emergency services, which may underestimate self-harm managed at home; incorporating the trauma/CSA checklist data described above into future survival models, once coded and validated, is a priority for follow-up analyses (19, 43).

### 4.3 Future research directions

Future research should utilize larger, multicenter cohorts with independent structured diagnostic confirmation of BPD to validate these findings and improve the generalizability of the interaction model. Additionally, extending the follow-up period beyond 12 months would allow researchers to examine the persistence of these clinical risk factors and identify emerging protective factors that may facilitate long-term stability and “recovery” from suicidal behavior in individuals with BPD traits. Future studies should also incorporate standardized assessments of social support, chronic stress, trauma exposure, and dimensional anxiety and mixed-affective symptoms, and should consider sensitivity analyses excluding bipolar-spectrum presentations.

## 5 Conclusion

In this exploratory secondary analysis, recurrent suicidal behavior among individuals with probable borderline personality disorder was associated with acute depressive severity and with a statistically significant, though imprecisely estimated, interaction between lifetime mood and anxiety-related disorders, independent of sociodemographic factors. Given the observational design, screening-based case ascertainment, and residual uncertainty around the interaction estimate, these findings are hypothesis-generating rather than conclusive. They support closer monitoring of depressive symptoms and comorbid affective-anxiety conditions as part of post-crisis risk stratification and secondary suicide prevention in this high-risk population, and active assessment and evidence-based management of depressive and anxiety-related symptoms, pending replication in larger, prospectively diagnosed samples.

## Data Availability

The raw data supporting the conclusions of this article will be made available by the authors, without undue reservation.
